# The Use of Umbelliferone in the Synthesis of New Heterocyclic Compounds

**DOI:** 10.3390/molecules16086833

**Published:** 2011-08-10

**Authors:** Ahmed A. Al-Amiery, Ahmed Y. Musa, Abdul Amir H. Kadhum, Abu Bakar Mohamad

**Affiliations:** 1 Department of Chemical and Processing Engineering, Faculty of Engineering and Built Environment, University of Kebangsaan Malaysia, Bangi, Selangor 43600, Malaysia; 2 Biotechnology Division, Applied Science Department, University of Technology, Baghdad 10066, Iraq

**Keywords:** coumarin, ethyl bromoacetate, phosphorous oxychloride, 1,3,4-thiadiazole, thiosemicarbazide, umbelliferone

## Abstract

New coumarin derivatives, namely 7-[(5-amino-1,3,4-thiadiazol-2-yl)methoxy]-2*H*-chromen-2-one (**4**), 5-[(2-oxo-2*H*-chromen-7-yloxy)methyl]-1,3,4-thiadiazol-2(3*H*)-one (**5**), 2-[2-(2-oxo-2*H*-chromen-7-yloxy)acetyl]-N-phenylhydrazinecarbothioamide (**7**), 7-[(5-(phenylamino)-1,3,4-thiadiazol-2-yl)methoxy]-2*H*-chromen-2-one (**8**) and 7-[(5-mercapto-4-phenyl-4*H*-1,2,4-triazol-3-yl)methoxy]-2*H*-chromen-2-one (**9**) were prepared starting from the natural compound umbelliferone (**1**). The newly synthesized compounds were characterized by elemental analysis and spectral studies (IR, ^1^H-NMR and ^13^C-NMR).

## 1. Introduction

Lactones constitute a large and diverse group of biologically active plant chemicals that have been identified in several plant families [1,2,3]. Coumarin and its derivatives represent one of the most active classes of compounds, possessing a wide spectrum of biological activity [4,5,6,7]. Many of these compounds have proved to be active as antibacterial [8,9], antifungal [10], anti-inflammatory [11], anticoagulant [12], anti-HIV [13] and antitumor [14] agents. Coumarins also have superior thermal stability and outstanding optical properties, including an extended spectral response, high quantum yields and superior photostability. Optical applications of these compounds, such as laser dyes, nonlinear optical chromophores, fluorescent whiteners, fluorescent probes, polymer science, optical recording and solar energy collectors, have been widely investigated [15,16,17,18,19]. The chemistry of thiosemicarbazones has received considerable attention because of their variable bonding modes, promising biological implications, structural diversity, and ion-sensing ability [20,21,22]. Thiazolidinones substituted in the 2-position, its derivatives and analogues exhibit unusually high *in vitro* activity against *Mycobacterium tuberculosis* [23,24,25]. In the current study we aimed to synthesize some new coumarins derived from umbelliferone (7-hydroxycoumarin) and thiazoles, with predictable biological activities. The chemical structures of the synthesized compounds were proven by IR, NMR spectra and elemental analysis data. 

## 2. Results and Discussion

### 2.1. Chemistry

**Scheme 1:**
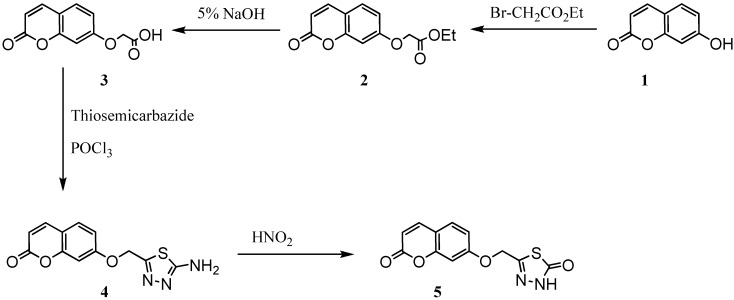
Synthesis of compounds 2–5.

**Scheme 2:**
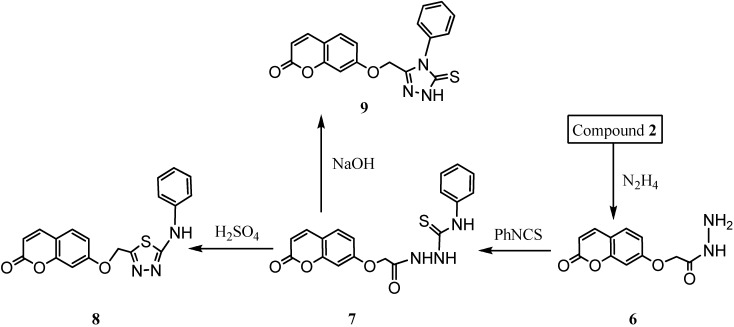
Synthesis of compounds 6–9.

For the synthesis of new umbelliferone derivatives, the reaction sequences outlined in Scheme 1 and Scheme 2 were followed. We started from umbelliferone (**1**) which is commercially available or, alternatively, readily accessible through a Pechmann and Perkin condensation [26]. Synthesis of ethyl 2-(2-oxo-2H-chromen-7-yloxy) acetate (**2**) was brought about by refluxing ethyl bromoacetate with umbelliferone in the presence of anhydrous K_2_CO_3_ in dry acetone. Reaction of compound **2** with sodium hydroxide yield compound 2-(2-oxo-2H-chromen-7-yloxy)acetic acid (**3**) that was cyclized with thiosemicarbazide in the presence of phosphorous oxychloride to yield 7-[(5-amino-1,3,4-thiadiazol-2-yl)methoxy]-2H-chromen-2-one (**4**) that was converted into compound **5** by adding sodium nitrate and hydrochloric acid. 

The addition of hydrazine to compound **2** yield compound **6** that was converted to compound **7** by addition of phenyl isothiocyanate. The cyclization of compound **7** by sulphuric acid yielded compound **8**, while reaction with sodium hydroxide produced compound **9**. 

In the IR spectrum of ethyl 2-(2-oxo-2H-chromen-7-yloxy)acetate (**2**) the lactone carbonyl stretching frequency was observed at 1759 cm^−1^, whereas the ester carbonyl stretching appeared at 1717 cm^−1^, and a C-H aliphatic stretching frequency appeared at 2987 cm^−1^that was not seen in the IR spectrum of the starting material umbelliferone, in addition of the disappearance of the hydroxyl group. In the ^1^H-NMR spectrum of compound **2**, a 3H *triplet* was observed at 3.126 ppm due to the ester CH_3_ protons and a *quartet* at 3.81 ppm due to the ester CH_2_ protons. The isolated CH_2_ protons were observed downfield as a singlet (2H) at 4.770 ppm and *triplet* (2H) at 5.260, 5.239 and 5.27. The coumarin alkene C-H appeared at 5.416 ppm. The protons of the aromatic ring were observed as *quartet* (7.472, 7.457, 7.413, 7.216 ppm for C_6_-H), *quintet* (7.565 7.543, 7.537, 7.525, 7.519 ppm for C_5_-H) and *doublet*, 7.895 ppm, 7.871 ppm for C8-H. The ^13^C-NMR spectrum analysis for compound **2**, combined with the information from ^1^H-NMR experiments, can be considered enough to guide future synthetic work. 

In the IR spectrum of 2-(2-oxo-2H-chromen-7-yloxy)acetic acid (**3**) the lactone carbonyl stretching frequency was observed at 1755 cm^−1^, whereas the carboxylic acid carbonyl stretching appeared at 1724 cm^−1^, and the hydroxyl group appeared as a broad band at 2975–3170 cm^−1^. In the ^1^H-NMR spectrum, the CH_2_ protons were observed downfield as a singlet (2H) at 4.83 ppm, and *triplet* (2H) at 5.311, 5.3 and 5.28. The C-H (alkene) of coumarin appeared at 5.418 ppm. The protons of the aromatic ring were observed as *quartet* (7.47, 7.455, 7.411, 7.207 ppm for C_6_-H), *triplet* (7.555 7.531, 7.527 ppm for C5-H) and *doublet* (7.90 ppm, 7.876 ppm) for C8-H. The ^13^C-NMR spectrum analysis for compound **3**, combined with the information from ^1^H-NMR experiments, can be considered enough to prove the structure of compound **3**. 

The IR spectrum is good evidence for formation of compound **4**. The absence of a hydroxyl group at 2975–3170 cm^−1^, and appearance of a new band at 3302 and 3343 cm^−1^, and a lactone carbonyl stretching frequency at 1749 cm^−^^1^ were observed. In the ^1^H-NMR spectrum, a singlet (thiadiazole-NH_2_) at 4.94 ppm and the C-H (alkene) of the coumarin appeared at 5.76 ppm and 5.891. The protons of the aromatic ring were observed as *multiplet* (7.51–7.28). In the ^13^C-NMR, 163.5 and 176.4 were new and due to the heterocyclic ring carbons. 

The IR spectrum of compound **5 **showed a carbonyl group at 1713 cm^−1^, in addition to an amino group at 3297 cm^−1^. In the ^1^H-NMR; the coumarin C-H (alkene) protons appeared at 5.81 ppm (*singlet*) and 6.43 ppm *(singlet*) and the *singlet* (NH) at 5.31 ppm. In the ^13^C-NMR the thiadiazole ring carbonyl carbon atom appears at very low field (171.2). 

**Scheme 3:**
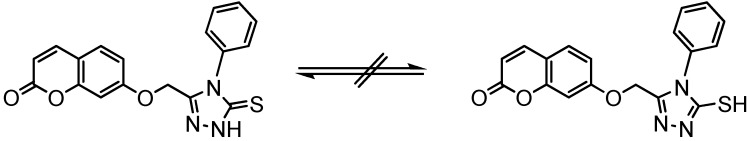
Tautomerization of thione.

Hydrazinolysis of compound **2 **with hydrazine hydrate afforded 2-(2-oxo-2H-chromen-7-yloxy)acetohydrazide (**6**) in good yield. The IR spectra of compound **6 **showed absorption bands in the 3351.3, 3287.1 cm^−1^ region (hydrazide NH-NH_2_), 1689.2 cm^−1^ (amide-C=O carbonyl stretching), and 1761.5 cm^−1^ (lactone-C=O carbonyl stretching). The ^1^H-NMR spectrum exhibited a *singlet* due to the –CO-NH-NH_2_ proton at δ 7.93 ppm. For compound **7**, the IR spectrum has the following characteristic absorption bands: ν_N-H_ (3367.6, 3301.2, 3278.9 cm^−1^); ν_C=O_ (1763 lactone; 1692.7 amide carbonyl cm^−1^), ν_C=S_ (1258.5 cm^−1^). In the IR spectrum of compound **8**, no absorption band at 1692.7 cm^−1^ was detected, indicating the absence of the amide carbonyl group, which is evidence for the conversion of compound **7** to compound **8**. Also, in the IR spectrum of the new heterocyclic compound **8** a stretching band characteristic of the C=N group from the thiadiazole nucleus appeared at 1620.6 cm^−1^. Although two types of tautomers, thione or thiole, could be expected from the cyclization of compound **7**, under basic conditions, only the thione type compound **9** was observed. The existence of the thione form predominantly in the solid state is demonstrated by the presence of two absorption bands at 1257 cm^−1^ and 3389.3 cm^−1^ belonging to the ν_C=S_ and ν_NH_ groups, respectively, and by absence of ν_SH_. In the ^13^C-NMR spectra of new heterocyclic compounds **8** and **9** the absence of the signals for the ester carbonyl and the absence of a thiocarbonyl carbon in compound **8**, confirmed that cyclization of compound **7** took place. In compound **9** the signal at 168.4 ppm indicated that in solution this compound exists predominantly in the thione tautomeric form (Scheme 3).

### 2.2. Computational Studies

#### 2.2.1. Atomic Charges and Stabilities

**Figure 1:**
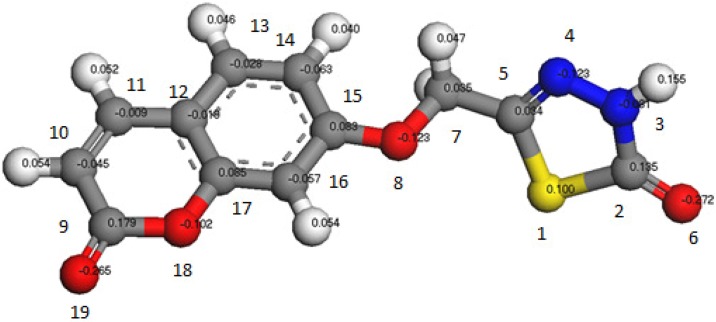
3d-geometrical structure for compound **5**.

The theoretical studies for compound **5** revealed that the atomic charges have been affected by the presence of the ring substituent. The minimized geometry is shown in Figure 1, where the calculated atomic charges for the compound are also indicated. It can be seen from Figure 1 that the highest atomic charge is at [O(6) −0.272)] the next charge value is at [O(19) −0.265]. These results clearly indicated that these two atoms are the most reactive sites toward the reactions and bonding with the metals. The calculated bond and twist angles (supplementary file) and 3d-geometrical structure, indicated that this molecule is not planar.

**Figure 2:**
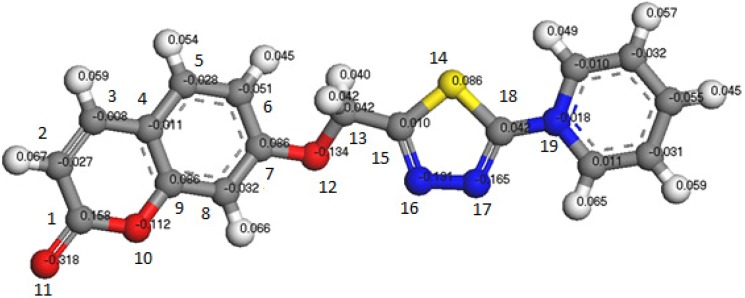
3d-geometrical structure for the compound **8**.

The minimized geometry for the compound **8** is shown in Figure 2. The calculated atomic charges for the compound are shown in the figure too. The results showed that the highest atomic charge is at [O(6) −0.272)] and the next charge value is at [O(19) −0.265]. These results showed clearly that these two atoms are the most reactive sites toward the reactions and bonding with the metals. The determined bond angles, twist angles and 3d-geometrical structure, indicate that this molecule is not planar and the C(2)-C(3) stereochemistry is (*Z*).

#### 2.2.2. Density Function Theory (DFT)

**Figure 3:**
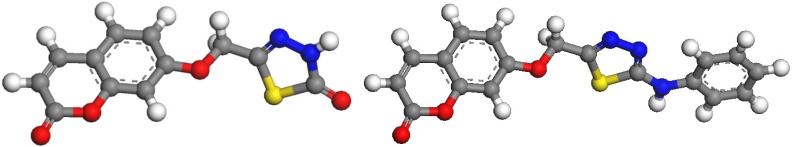
Optimized molecular structures of **5** and **8** by DFT.

**Figure 4:**
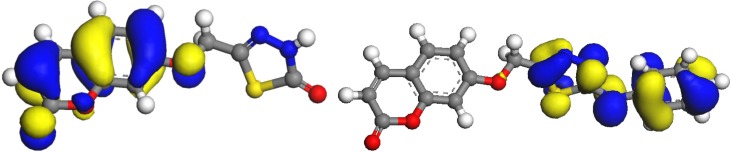
HOMO orbitals of **5** and **8**.

DFT calculations were performed for compounds **5** and **8**. Optimized molecular structures of the most stable forms are shown in Figure 3. Their calculated energies and relative energies are presented in Table 1. Molecular orbital calculations provide a detailed description of orbitals including spatial characteristics, nodal patterns and individual atom contributions. The contour plots of the frontier orbitals for the ground state of **5** and **8** are shown in Figure 4, including the Highest Occupied Molecular Orbital (HOMO) and the Lowest Unoccupied Molecular Orbital (LUMO). It is interesting to see that both orbitals are substantially distributed over the conjugation plane. It can be seen from the Figure 4 that the HOMO orbitals are located on the substituted molecule while LUMO orbitals resemble those obtained for the unsubstituted molecule and therefore the substitution has an influence on the electron donation ability, but only a small impact on electron acceptance ability [27]. The orbital energy levels of HOMO and LUMO of compounds **5** and **8** are listed in Table 2. It can be seen that the energy gaps between HOMO and LUMO is about 0.11 and 0.008 H.a. for the compounds **5** and **8**, respectively. The lower value in the HOMO and LUMO energy gap explain the eventual charge transfer interaction taking place within the molecules. The dipole moments of compounds **5** and **8**, were also calculated and listed in Table 3. The dipole moment for compounds **5** is oriented inwards, while the dipole moment for compounds **8**, is oriented outwards.

**Figure 5:**
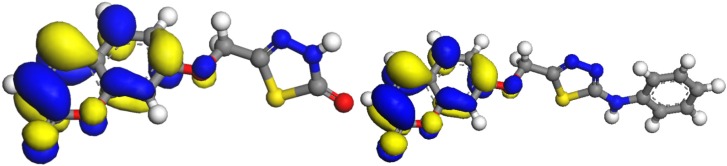
LUMO orbitals of **5** and **8**.

**Table 1:** Total Energy, relative energies and heat of formation (H.a.) for **5** and **8**.

	Total energy	Sum of atomic energies	Kinetic	Electrostatic	Binding energy
5	−120.394	−1265.697	−11.406	2.934	−5.061
8	−145.743	−1474.474	−11.317	−0.569	−7.135

**Table 2:** HOMO and LUMO energies of **5** and **8**.

	HOMO (H. a)	LUMO (H. a)	∆E
5	−0.207	−0.097	−0.11
8	−0.190	−0.092	−0.098

**Table 3:** The dipole moments (Debye) of **5** and **8**.

	x-component	y-component	z-component	magnitude
5	−0.11	2.694	0.019	2.697
8	2.016	4.254	0.627	4.748

## 3. Experimental

### 3.1. General

The chemicals used for the synthesis were supplied by Sigma-Aldrich. Purity of the compounds was checked on thin layer chromatography (TLC) plates (Silicagel G) using the solvent systems benzene-ethyl acetate-methanol (40:30:30, v/v/v) and toluene-acetone (75:25, v/v). The spots were located under UV light (254 and 365 nm). The IR spectra were obtained on a Thermo Scientific, Nicolet 6700 FT-IR spectrometer (without KBr or CsI pellets). The ^1^H-NMR spectra were obtained on a Jeol jnm-ECP400 FT-NMR system. Elemental microanalysis was carried out using a model 5500-Carlo Erba C.H.N elemental analyzer instrument. A Gallenkamp M.F.B.600.010 F melting point apparatus was used to measure the melting points of all the prepared compounds.

### 3.2. Synthesis

*Ethyl 2-(2-oxo-2H-chromen-7-yloxy)acetate* (**2**). A suspension of 7- hydroxycoumarin (6.17 mmol) in acetone (30 mL) was refluxed with ethyl bromoacetate (9.15 mmol) and K_2_CO_3_ (4.69 g, 33.91 mmol) for 12 h. After cooling, the mixture was evaporated to dryness and the residue was partitioned between CHCl_3_ (50 mL) and water (50 mL). The organic phase was dried (Na_2_SO_4_), filtered and evaporated to dryness. The residue was recrystallized from acetone, yield 82%, m.p. 112.5 °C; ^1^H-NMR (CDCl_3_): *δ* 3.126 (t, 3H, CH_3_), *δ* 3.81 (m, 2H, CH_2_), *δ* 4.77 (s, 2H) and *δ* 5.260, *δ* 5.239, *δ* 5.270 (s, 2H, CH_2_), *δ* 5.416 (s, 1H, –C=C-H), *δ* 7.472, *δ* 7.457, *δ* 7.413, *δ* 7.216 (q, 1H, aromatic); ^13^C-NMR (DMSO-d_6_): 22.3, 58.9, 65.1, 103.8, 108, 108.9, 110.1, 123.7, 154.6, 157.1, 160.8, 161.2, 166.5; IR: 2987 cm^−^^1^ (C-H, aliphatic), 1759 cm^−^^1^ (C=O, lactone), 1717 cm^−^^1^ (C=O, ester); Anal. Calcd. for C_13_H_12_O_5_: C 62.90%, H 4.87%. Found: C 61.81% H 4.01%.

*2-(2-Oxo-2H-chromen-7-yloxy)acetic acid* **3**. A solution of the compound **2** (2.7 mmol) and 5% sodium hydroxide (2.16 mL) in ethanol (15 mL) was stirred under reflux for 2 h. After removal of the solvent, the residue was dissolved in water and acidified with HCl 6 M. The white solid collected by filtration was washed with cool water, dried and recrystallized from ethanol, yield 92%, m.p. 210.5 °C; ^1^H-NMR (CDCl_3_): *δ* 4.83 (s, 2H) and *δ* 5.311, *δ* 5.300, *δ* 5.280 (s, 2H, CH_2_), *δ* 5.418 (s, 1H, –C=C-H), *δ* 7.470, *δ* 7.455, *δ* 7.411, *δ* 7.207 (q, 1H, C_6_-H aromatic ring), *δ* 7.555, *δ* 7.531, *δ* 7.527 (t, 1H) for C_6_-H aromatic ring, *δ* 7.90, *δ* 7.876 (s, 1H, C_8_-H aromatic ring ); ^13^C-NMR (DMSO-d_6_): 105.2, 110.3, 112.9, 113.9, 124.1, 153.9, 156.3, 161.2, 161.9, 167.1; IR: 3170-2975 cm^−1^ (hydroxyl), 1755 cm^−^^1^ (C=O, lactone), 1724 cm^−1^ (C=O, carboxylate); Anal. Calcd. for C_11_H_8_O_5_: C 60.00%, H 3.66%. Found: C 58.40% H 2.94%.

*7-[(5-Amino-1,3,4-thiadiazol-2-yl)methoxy]-2H-chromen-2-one* (**4**). Phosphorus oxychloride (20 mL) was added to compound **3** (0.05 mol) and the mixture was stirred for 1 h. at room temperature. Thiosemicarbazide (4.56 gm, 0.05 mol) was added and the mixture was heated under reflux for 5 h. On cooling, the mixture was poured on to ice. After 4 h. the mixture was stirred for 15 min. to decompose the excess phosphorus oxychloride, then heated under reflux for 30 min, cooling, the mixture was neutralized by 5% potassium hydroxide, the precipitate was filtered, washed with water, dried and recrystallized from ethanol, yield 51%, m.p. 105 °C; ^1^H-NMR (CDCl_3_): *δ* 4.94 (s, 1H, NH_2_), *δ* 5.760, 5.891 (s, 1H, –C=C-H), *δ* 7.510–7.283 (m, 1H, C-H aromatic ring); ^13^C-NMR (DMSO-d_6_): 101.9, 111.0, 113.1, 114.1, 128.1, 149.5, 156.3, 160.8, 161.2, 163.5, 168.3, 176.4; IR: 3302 and 3343 cm^−^^1^ (N-H, amine), 1747 cm^−^^1^ (C=O, lactone); Anal. Calcd. for C_12_H_9_N_3_O_3_S: C 52.36%, H 3.30%, N 15.26%. Found: C 50.49% H 2.76%, N 16.20%.

*5-[(2-Oxo-2H-chromen-7-yloxy)methyl]-1,3,4-thiadiazol-2(3H)-one* (**5**). 10% Aqueous sodium nitrite solution (10 mL) was added dropwise with continuous stirring over a period of 20 min to an cooled (ice-bath) suspension of compound **4** (0.01 mol) and hydrochloric acid (5 mL) in cold water (20 mL). The temperature was then allowed to rise to room temperature and the mixture was heated to boiling for 10 min, cooled and allowed to stand overnight. The separated crude product was filtered, washed with water, dried and recrystallized from ethanol, yield 47%, m.p. 133 °C; ^1^H-NMR (CDCl_3_): δ 5.31 (s, 1H, NH), δ 5.810, 6.430 (s, 1H, –C=C-H), δ 7.811–7.299 (m, 1H, C-H aromatic ring); ^13^C-NMR (DMSO-d_6_): 103.2, 115.1, 117.5, 117.9, 123.2, 155.3, 155.9, 156.4, 160.8, 161.1, 166.2, 171.2; IR: 3397 cm^−1^ (N-H, amine), 1713 cm^−1^ (C=O, lactone); Anal. Calcd. for C_12_H_8_N_2_O_4_S: C 52.17%, H 2.29%, N 10.14%. Found: C 50.34% H 3.01%, N 9.51%.

*2-(2-Oxo-2H-chromen-7-yloxy)acetohydrazide* (**6**). A solution of compound **2 **(10 mmol) in ethanol (25 mL) was refluxed with hydrazine hydrate (25 mmol) for 4 h. After concentrating the reaction mixture a solid mass separated out and was recrystallized from ethanol, yield 75%, m.p. 234.5 °C; ^1^H-NMR (CDCl_3_): *δ* 7.93 (-CO-NHNH_2_), *δ* 4.61 (s, 2H) and *δ* 5.251, *δ* 5.222, *δ* 5.214 (s, 2H, CH_2_), *δ* 5.716 (s, 1H, –C=C-H), * δ* 7.61–7.110 (s, 1H, aromatic ring); IR: 3351.3 and 3287.1 cm^−1^ (N-H, hydrazide), 1761.5 cm^−1^ (C=O, lactone), 1689.2 cm^−1^ (C=O, amide); Anal. Calcd. for C_11_H_10_N_2_O_4_: C 56.40%, H 4.38%, N 11.96%. Found: C 54.44%, H 4.04%, N 10.79%.

*2-[2-(2-Oxo-2H-chromen-7-yloxy)acetyl]-N-phenylhydrazinecarbothioamide* (**7**). A mixture of hydrazide **6** (2 mmol) and phenyl isothiocyanate (2 mmol) in ethanol (15 mL) was refluxed for 12 h. The reaction mixture was cooled and the separated product was filtered off, dried and recrystallized from ethanol, yield 61%, m.p. 199 °C; IR: 3367.6, 3301.2 and 3278.9 cm^−1^ (N-H, hydrazone), 1763 cm^−1^ (C=O, lactone), 1692.7 cm^−1^ (C=O, amide), 1258.5 cm^−1^ (C=S); Anal. Calcd. for C_18_H_15_N_3_O_4_S: C 58.53%, H 4.09%, N 11.38%. Found: C 56.49%, H 3.78%, N 10.22%. 

*7-[(5-(Phenylamino)-1,3,4-thiadiazol-2-yl)methoxy]-2H-chromen-2-one* (**8**). A mixture of compound **7** (0.15 mmol) and concentrated H_2_SO_4_ (5 mL) was stirred in ice path for 5 h and then, at room temperature, for another 5 h. The reaction mixture was neutralized with a diluted solution of ammonium hydroxide, in ice bath. The precipitated was filtered off, washed with water, dried and recrystallized from ethanol, yield 57%, m.p. 244 °C; IR: 3291 cm^−1^ (N-H), 1762.8 cm^−1^ (C=O, lactone), 1620.6 cm^−1^ (C=N); ^13^C-NMR (DMSO-d_6_): 100.3, 107.2, 111.9, 114.2, 122.0, 125.4, 126.2, 126.8, 129.2, 129.7, 141.6, 156.2, 156.9, 157.3, 158.1, 160.5, 169.3; Anal. Calcd. for C_18_H_13_N_3_O_3_S: C 61.53%, H 3.73%, N 11.96%. Found: C 61.01%, H 3.11%, N 11.32%.

*7-[(4-Phenyl-5-thioxo-4,5-dihydro-1H-1,2,4-triazol-3-yl)methoxy]-2H-chromen-2-one* (**9**). Compound **7** (0.5 mmol) was added to 8% NaOH solution (4 mL) and the reaction mixture was heated under reflux for 4 hours. After cooling, the solution was acidified with a diluted solution of HCl. The crude product was precipitated, filtered off and washed with water. The solid thus separated was dried and recrystallized from chloroform, yield 44%, m.p. 251 °C; IR: 3389.3 cm^−1^ (N-H), 1767.7 cm^−1^ (C=O, lactone), 1618.1 cm^−1^ (C=N), 1257 cm^−1^ (C=S); ^13^C-NMR (DMSO-d_6_): 99.6, 109.8, 112.3, 114.1, 122.3, 127.1, 129.3, 129.8, 130.1, 130.9, 134.4, 154.0, 155.8, 156.5, 157.2, 163.6, 168.4; Anal. Calcd. for C_18_H_13_N_3_O_3_S: C 61.53%, H 3.73%, N 11.96%. Found: C 61.01%, H 3.11%, N 11.32%.

### 3.3. DFT

The molecular drawings of the nine thio compounds were plotted using Visualization Materials Studio 5.5. All quantum chemical calculations were performed using Density Functional Theory (DFT) as implemented in the Materials Studio 5.5 software. DMol^3^ model was employed to obtain quantum chemical parameters and to optimize the molecules’ geometry. These calculations employed an *ab initio*, generalized gradient approximation (GGA) with the Lee-Yang-Parr correlation functional (BLYP) functional and Double Numerical d-functions (DND) basis set. This approach is shown to yield favorable geometries for a wide variety of systems. The following quantum chemical indices were calculated: the energy of the highest occupied molecular orbital (HOMO), the energy of the lowest unoccupied molecular orbital (LUMO) and dipole moment. 

## 4. Conclusions

In this study, the compounds **2–9** were synthesized, and characterized using various spectroscopic methods and elemental analysis. The synthesized compounds were studied theoretically and the atomic charges, heat of formation and stereochemistry were estimated, and it was found that compounds **5** and **8** are not planar.
